# Medical Miscellanies

**Published:** 1816-04

**Authors:** 


### PART IV.

### MEDICAL MISCELLANIES.

### ELECTRICITY.

The result of my inquiries into the curative merits of Electricity, tends to confirm the prevailing opinion of my professional brethren, that it appears more specifically useful in rheumatic complaints than in any other morbid affections.

Reasoning from the success that has attended its administration (and the experimental tests instituted by me have not been circumscribed), I consider myself enabled to recommend it as a valuable appendage to the healing art, as one that will often answer in the above complaints when other means fail, and as one that deserves the notice of the Faculty.

In looking over my case-book for satisfactory proofs of this truth, I find that not merely the majority of my patients afflicted with chronic rheumatism have been cured, but that I am unable to adduce more than two cases which have withstood its salutary influence. In these, the shoulder joint was the part affected. One of them was a poor woman, aged 50 years, who had been for several months under the care of a physician in the city, and who failing in his attempts to perform a cure by medical agency, did me the honour to place her under my care in 1813. The other was a gentleman about 35 or 40 years of age, who, after exhausting the materia medica, was recommended by his surgeon-apothecary to give electricity a trial, and applied to my late friend, Mr. Birch, one of the surgeons to St. Thomas's Hospital, for assistance. In consequence of Mr. B's impaired state of health, this gentleman, with his other patients, was transferred to me. I administered electricity, in every devisable form, to both these cases, three or four times a week for two months, with no other benefit than a partial remission of the pain, for the rigidity of the parts surrounding the shoulder-joint and scapula still remained.

These patients could not be prevailed upon to continue the use of electricity longer than the above period, and the want of success gave me considerable disappointment. The woman I have not seen since; but the gentleman, a few months afterwards, acquainted me, that the change in the weather, produced by the advancement of summer, had entirely effectuated his recovery. The last five months have furnished me with fourteen rheumatic cases, to twelve of which the electric fluid has been used specifically; the other two are still my patients, and are progressively mending. In some the disease had been of three or four weeks' continuance; in others, the same number of months; and in a few, almost as many years. Most of them had been treated by all the medicaments usually resorted to with advantage, without receiving any other benefit than temporary relief, and came to TRY electricity as a dernier resort.

In recent cases, I have found the electric sparks drawn from the parts affected, when insulated, for the space of a quarter of an hour every other day, and continued till the evanescence of all unpleasant symptoms, fully adequate for the removal of rheumatism: but in the more obstinate cases, this form cannot be considered so efficacious as shocks. In these, my plan is to commence the cure by passing fifteen or twenty shocks slightly through the diseased part, and gradually augmenting their force and number according to the feelings of the patient, till the pain and rigidity of the parts are removed.

The benefits arising from electricity are sometimes almost instantaneous, and the patient is overjoyed to find a power of motion in the parts affected, after the first electrization, which he did not before possess. This effect, however, though we are to deem it a proof of the salutary influence of our remedy, ought to be esteemed only as transient; for unless the electrical administration be persisted in, and even repeated a few times after all appearances of the complaint are removed, the cure is most assuredly to be considered as incomplete. Many feel no advantage till electricity has been used for some time; and even then their recovery is so progressive, that they are inclined to impute their amendment to any other cause than the benign operations of this useful but painful remedy. One or two omissions, however, of the accustomed attendance upon the machine, convince them of their mistake, and by enabling the disease to gain such an ascendancy, that several subsequent electrizations are required to compensate for the neglect, at last teach them to ascribe the gradual relief they have experienced to its right source.

In very obstinate cases, and those which have resisted the powerful influence of diaphoretics, stimulants, tonics, warm clothing, and change of seasons, as is often seen when the complaint is seated in the shoulder joint, electricity must be persisted in, on most occasions, for weeks, and even months, till success is the eventual result. A case of this kind is now under my care: the patient is a respectable tradesman residing in Soho; he has been afflicted with rheumatism upwards of two years in the arms and shoulders; and, although the remedy has at present made but little impression during the three weeks which I have used it, I can, without empirical pretension, assure him, that his complaint is in the power of electricity to remove.

But to insure success, it is indispensible to act fairly with the remedy, by persisting regularly in its use. Should the patient not conform to this direction, he will be alone to blame, and any imputation on the virtues of electricity would be uncandid and unjust. I have often cause to regret, that patients are too inattentive in this particular, and that as soon as all the urgent symptoms are removed, they will absent themselves till they are compelled to return by a recurrence of the pain. This is the best explanation I can give for having patients upon my list four or five months, when they could have been completely cured by a steady perseverance on their parts in as many weeks.

The only medical adjuvants which I find it necessary to prescribe with electricity are of the aperient class, and I inculcate the propriety of strict attention to the non-naturals.

Atmospherical change, and successive alternations of dryness and moisture, materially affect rheumatic patients; they impede electrical efficacy, and render it far more tedious than the most lasting cold weather, accompanied either with settled dryness or long-continued humidity.

To this testimony of the salutary effects of electricity, I am happy to add that of a gentleman, whose experience of its operations is greater than my own. Mr. Carpue tells me he has a list of upwards of three hundred well-marked rheumatic cases which he formerly cured by electricity, when he employed this branch of the healing art extensively in his practice.

BENJAMIN GOLDING.
13, Leicester Place, Leicester Square.

*Extract of a Letter from Mr. H. W. Bailey to Dr. Shearman, dated Thetford, March* 12, 1816.—I was pleased with the Communication of Mr. Johnson, under Vaccination, in the fourth department of your Journal, wherein he recommends the insertion of the point armed with vaccine lymph. I can speak from experience, that with this mode I have vaccinated many hundred children, and have never had occasion to re-vaccinate one. When many are required to be vaccinated at one time, as generally happens in parishes, I have well moistened a piece of thread, which, when dry, is cut into lengths about one sixteenth part of an inch, and inserted under the cuticle, having made the incision in the way adopted by Mr. Johnson. A piece of adhesive plaster is then applied over the vaccinated part, which may be removed a day or two after. So doubtful was the security of vaccination looked upon, in 1813, that I paid the greatest attention to the progress of this mild disease. In every case, I re-vaccinated them on the fifth day from the first vaccination; and invariably found the second inoculation arrive to the same state of maturity at the time the first did: thus proving the accuracy of Mr. Bryce's test. These patients were thrice variolated by an old woman, and in no one instance was small-pox produced.

---

*Extract of a Letter from Dr. James Kennedy to Dr. Palmer, dated Dunning, Feb. the 7th,* 1816.—— "Some slight omissions have been made in the case of poisoning by nitric oxide of quicksilver, which I lately communicated to you for insertion in your Journal. I have seen the man since I wrote to you: he is in good health, but informs me that he is losing almost all his hair. It has also been omitted to say, that on the second day (Monday) a copious ptyalism appeared, and did not entirely subside in less than a fortnight. Will you have the goodness to note these omissions in any way you may think proper?"

---

*Case of Umbilical Tumor, communicated by a Correspondent to Dr. Palmer.*——A male child, aged about four months, was brought to my house last September, having his umbilicus covered with a pendulous tumor, equal in size to a pullet's egg; which originated by a neck considerably thicker than the barrel of a common quill: its parietes were thin, elastic, and beautifully vascular, and contained a thinnish fluid. The structure of the neck was less exquisite and refined. This tumour had made its first appearance, six weeks previously, by a slight elevation of the navel, and rapidly increased to the present size. From an idea that the case was umbilical hernia, compression had been employed: but the child soon exhibited strong marks of uneasiness, and the bandage was removed. The umbilical cord, at the child's birth, had been divided, and afterwards sloughed off, in the usual manner. All the internal functions were regularly performed; the phænomena of health strongly marked and predominant. The vascular structure of the membranous sac suggested a dread of hæmorrhage in removing it with the scalpel. Ligature, therefore, was preferred: and the neck of the tumour was tightly begirt with a waxed silken thread. I did not again see the child for more than a month. But the mother informs me that, on the second day from the application of the ligature, the vascular rays of the sac became faded and black; that it acquired a dry shrivelled aspect on the third; and on the fourth, dropped off. It contained nearly a tea-spoonful of fluid, yellowish in colour, and of the consistence of a syrup. The umbilicus has now completely regained its natural form.

*Singular consequences of an operation for Castration.*——A stout young man, aged twenty-six, affected from infancy with inguinal hernia on the left side, had a large, hard, heavy tumour in the scrotum on the corresponding side. It was separated from the abdominal ring by a distinct interval; yielded an obscure sense of fluctuation; and had commenced ten years before. The case was pronounced sarcocele; and the removal of the tumour decided upon. The hernia was small, and spontaneously disappeared on laying the patient in a recumbent posture. The operation was performed in the usual manner by an experienced surgeon, and the tumor extracted from its containing membranous cyst. But in dissecting the cord, the hernial sac, which had contracted adhesions to it, was opened; and the spermatic artery was found so much ramified that it was necessary to include the whole cord in a general ligature. Immediately after the dressing, considerable hæmorrhage took place from the wound, accompanied by extreme weakness, vomiting, and pain in the side of the neck. The hæmorrhage was repressed by piling linen and lint upon the wound, so as to effect a sort of compression.—Evening. Great tension of the abdomen; frequent eructation; pulse small and hurried; skin cold; incessant vomiting.—Second day. Abdominal tension increased; medicines immediately rejected; skin less cold.—Third day. Continued and exhausting vomitings, only allayed by white wine: mind restless and disturbed; violent colics. The ligatures were removed in the idea that from them originated the nervous symptoms: no hæmorrhage took place.—Fourth day. Five o'clock, A. M. slight amendment. Ten A. M. Face shrunk; skin cold; weakness excessive. Three, P. M. Death: all the intellectual faculties undisturbed.—*On Dissection*, traces of slight peritoneal inflammation were discovered: the intestines covered with a thin layer of albumine, adhered together. The abdominal cavity contained *more than a pint of black blood mixed with coagula*: it had penetrated into the hypochondria, beneath the liver and around the spleen; and filled the pelvis. The patient then died from effusion of blood into the abdomen, which had penetrated by the opening of the hernial sac, formed during the operation, and when the hæmorrhage took place, for which compression was employed. On examining the tumour, it was found not at all to implicate the testicle, which was sound, and situated below. It appeared to have been developed at the expence of the epididymis; and was a real cyst, the fibro-cartilaginous parietes of which, a line and a half in thickness, contained a reddish albuminous fluid, and a red brown pulpy substance resembling the scum formed by boiling blood in water. A sort of brownish false membrane lined the whole interior of the cyst, except a place corresponding to the *corpus highmorianum*, where several whitish ulcerated points existed. *Bulletin de la Société Médicale d'Emulation, Octobre*, 1815.

Mr. Johnson had lately an opportunity of opening the head of a boy, who died with symptoms of hydrencephalus. He was eleven or twelve years of age, and till within a week or ten days of his death, he had made little or no complaint. About five weeks before his decease, he had fallen down a ladder, and bruised the back part of his head. He did not however, take much notice of the accident; nor did he mention the circumstance for some time afterwards. The first morbid symptoms were derangement of the bowels; the fæces extremely fœtid; headache; subsequent strabismus; vomiting; constant jactitation, with irregularity of the pulse. The face was generally much flushed, and the vessels of the eye were distended with blood. The boy did not come under medical cognizance till symptoms of effusion had commenced; that is, about six days before death. Blisters to the head and neck; purgatives and mercurials, with digitalis, were employed, but to no effect. On dissection no apparent trace of inflammation or turgescence of vessels was visible. The substance of the brain was extremely soft; and about six ounces of water were effused into the ventricles. No organic injury was produced on the skull or membranes by the fall. Did the concussion lead to the effusion of fluid in the ventricles? Did not this effusion remove the turgesence or inflammation of the vessels?

*Apothecaries Bill.* — A very spirited memorial has been drawn up by "the Medical and Surgical Practitioners of Newcastle upon Tyne," and circulated pretty largely, calling on their brethren throughout the Kingdom, to unite "in an attempt to procure by petition, and every other legitimate means, a repeal or modification of the Act." The memorial enters minutely into the merits and demerits of the said Act, and certainly exposes its innumerable flaws and absurdities with great power of reasoning. We cannot afford space but for a very few extracts.

"It places in the hands of the Physician a power too indefinite, and by exhibiting the Surgeon-Apothecary in an abject capacity, it detracts from the creditableness of his calling, and consequently from his usefulness in society. It outrages those very feelings which the system of education is intended to generate and to cherish." The memorialists forbear to insist, on that disrespect for the Licentiates of the College of Physicians, as well as for the Graduates of all Universities, excepting Oxford and Cambridge, into which, the society of Apothecaries have, by their submission in this particular, suffered themselves to be betrayed." The inquisitorial inspection of Apothecaries shops, while Druggists and

Chemists are exempted from all domiciliary visits, is forcibly portrayed.

But the apprentices are the most aggrieved class. While the bill is prospective on quacks and unprincipled nostrum mongers, it is *retrospective* on apprentices ! — The term too of five years, so objectionable, especially in the country, where no lectures can be attended during the period of apprenticeship. "*The prima facia* severity of confining a youth for five years to a pestle and mortar in the shop of a country apothecary, at a distance from all scientific or academical education is so palpable, as loudly to call for the interposition of the legislature."

"Upon the whole, the memorialists, in bringing these observations near to a conclusion, shall remark, generally, that they have scarcely been able to find in the Act, a single clause, the advantages of which are not, to provincial practitioners, more than counterbalanced by the disadvantages —That its chief tendency, however opposite to the design of the original movers, appears to be, to open a door to the intrusion of *irregular pretenders* — to concentrate all the advantages in the Company of Apothecaries of London — to encourage a species of monopoly and exclusive right subversive of the purer principles of medical philosophy — to heap difficulties and degradations on the country practitioners, and to exclude them from all participation in those benefits to which their claim is as just and imprescriptible as that of their brethren in the metropolis."

Among the subscribers, appear John Ramsay, M. D. T. E. Headlam, M. D. Dr. Robert Stevenson — Dr. Thomas Trotter — Dr. W. S. Burne, &c. &c.

Dr. Burder has been elected Physician to the Westminster General Dispensary; and Mr. Babington Assistant-Surgeon to the Lock Hospital.

Dr. Adams is preparing for the press, Memoirs of the Life, Doctrines, and Opinions of the late John Hunter, founder of the Hunterian Museum, at the College of Surgeons in London. These memoirs are carefully collected from authentic documents and anecdotes, and also from the writings, lectures, and conversations of the deceased.

Deaths.—Dr. William Allen, lately attached to the Medical Staff at Paris.—In Gun Street, Spitalfields, Dr. William Cullen Brown, son of Dr. John Brown, the celebrated author of Elementa Medicina.—Mr. Alexander Petland, of Acton, Surgeon.

